# Exploratory Evaluation of Cardiac Fatty Acid-Binding Protein (H-FABP), Troponin I, NT-proBNP, Echocardiography, and Holter Monitoring for the Assessment of Doxorubicin-Induced Cardiac Damage in Dogs

**DOI:** 10.3390/vetsci13080735

**Published:** 2026-07-24

**Authors:** Paloma Nicolás-Barceló, Jesús Fortes-Díaz, Rafael Barrera, Pablo Cardenal-Morales, José Ignacio Cristóbal, Ángela Durán, Inmaculada Sevidane, Francisco Javier Duque

**Affiliations:** 1 MINVET Research Group, Departamento de Medicina Animal, Facultad de Veterinaria, Universidad de Extremadura, 10003 Cáceres, Spain; pnicolash@alumnos.unex.es (P.N.-B.); rabacha@unex.es (R.B.); jicristob@unex.es (J.I.C.); angeladg@unex.es (Á.D.); isevidan@alumnos.unex.es (I.S.); 2 Veterinary Teaching Hospital, Veterinary Faculty, University of Extremadura, 10003 Cáceres, Spain; jfortesd@gmail.com (J.F.-D.); pablocm@unex.es (P.C.-M.)

**Keywords:** doxorubicin, h-FABP, troponin I, cardiotoxicity, canines, Holter

## Abstract

Doxorubicin-induced cardiotoxicity is an important concern in veterinary oncology, and early detection of myocardial injury remains clinically challenging. This exploratory pilot study described changes in cardiac fatty acid-binding protein (h-FABP), cardiac troponin I (cTnI), NT-proBNP, conventional echocardiographic variables, and 24-h Holter-derived heart rate variability parameters in dogs with hemangiosarcoma treated with doxorubicin. Fourteen dogs were prospectively evaluated before treatment initiation and one week after the third doxorubicin dose, corresponding to a cumulative dose of 90 mg/m^2^. The study did not include a control group, a predefined cardiotoxicity endpoint, or diagnostic-performance analysis. Plasma biomarkers, echocardiographic parameters, ventricular arrhythmic events, and Holter-derived time- and frequency-domain heart rate variability variables were compared between both time points. Cardiac troponin I increased significantly after three doxorubicin doses, from a median of 0.05 ng/mL to 0.15 ng/mL (*p* = 0.002). In contrast, h-FABP did not change significantly, with median values of 2.43 ng/mL at baseline and 2.62 ng/mL after treatment (*p* = 0.27). NT-proBNP also showed no significant change (*p* = 0.87). Among echocardiographic variables, E-point septal separation, fractional shortening, ejection fraction, LA/Ao, and LVDd index did not change significantly. Ventricular arrhythmic events were infrequent and were described descriptively, whereas Holter-derived HRV variables did not show statistically significant differences between time points. In this exploratory cohort, cTnI increased after three doxorubicin doses, whereas h-FABP and NT-proBNP did not change significantly at the evaluated time point. Because h-FABP was measured one week after treatment, its potential short-term post-infusion release cannot be assessed. Larger studies with serial early sampling, predefined cardiotoxicity endpoints, appropriate control groups, and adjusted statistical analyses are required.

## Simple Summary

Doxorubicin is an effective chemotherapy drug used in veterinary oncology, but its use may be limited by the risk of cardiac injury. Detecting cardiac effects before overt heart dysfunction develops remains challenging. This exploratory study evaluated several blood-based and cardiac monitoring variables in dogs with hemangiosarcoma before doxorubicin treatment and one week after the third doxorubicin dose. Fourteen dogs were included, and cardiac biomarkers, echocardiography, and 24-h Holter monitoring were assessed. Cardiac troponin I increased after three doxorubicin doses, indicating a statistically detectable within-dog biomarker change at this time point. In contrast, h-FABP and NT-proBNP did not change significantly, and most echocardiographic and Holter-derived variables did not show statistically significant differences. Because h-FABP was measured one week after doxorubicin administration, this study cannot exclude a short-lived increase occurring earlier after infusion. These findings should therefore be considered preliminary and hypothesis-generating. Larger studies with serial early sampling after each doxorubicin administration and predefined cardiotoxicity endpoints are needed to clarify the clinical value of h-FABP and other monitoring tools in dogs receiving doxorubicin.

## 1. Introduction

Doxorubicin (DOX) is an anthracycline antibiotic widely used in veterinary oncology due to its high efficacy against a broad spectrum of hematopoietic and solid neoplasms, such as multicentric lymphoma, hemangiosarcoma, and various carcinomas [1]. Despite its indisputable therapeutic value, the use of doxorubicin is limited by notable systemic adverse effects, with cardiotoxicity being a serious and potentially irreversible long-term complication [2]. This toxicity limits the maximum cumulative dose that a canine patient can safely tolerate, frequently compromising the success of the overall chemotherapeutic protocol [3].

The pathophysiological mechanism underlying doxorubicin-induced cardiotoxicity is multifactorial, although the most widely accepted theory focuses on the induction of reactive oxygen species (ROS) production [3]. Doxorubicin exhibits a high affinity for cardiolipin, an abundant phospholipid located in the inner mitochondrial membrane of cardiomyocytes [4]. Upon binding, it dysregulates the electron transport chain, provoking severe mitochondrial dysfunction, lipid peroxidation of cellular membranes, and cytoplasmic vacuolation [5]. As cardiac tissue possesses limited antioxidant defenses (low levels of catalase and superoxide dismutase) compared to other organs, it is highly vulnerable to cumulative ROS-mediated damage. This injury progresses silently from subclinical myocardial lesions to secondary dilated cardiomyopathy, characterized by systolic dysfunction and refractory congestive heart failure [6].

Clinical monitoring of this condition has traditionally relied on conventional echocardiography [7]. Kinetic parameters such as fractional shortening (FS) and ejection fraction (EF) are routinely used in clinical practice to assess systolic function prior to each chemotherapy cycle. However, conventional echocardiography lacks adequate sensitivity to detect early myocardial injury. Changes in FS and EF typically manifest only when cellular damage is extensive and myocardial compensatory mechanisms have been overwhelmed, meaning that traditional echocardiographic diagnosis is often late and ineffective for preventing permanent structural changes [8].

On the other hand, continuous ambulatory electrocardiographic monitoring (Holter) has emerged as a non-invasive complementary tool for assessing cardiac autonomic status and detecting transient chemotherapy-induced arrhythmias [9]. The analysis of heart rate variability (HRV) via Holter allows for the evaluation of the sympathetic–parasympathetic balance exerted on the sinoatrial node. It has been classically postulated that anthracycline cardiotoxicity induces early dysautonomia, characterized by a decrease in HRV indices prior to the occurrence of macroscopic echocardiographic alterations, rendering Holter monitoring a potential candidate for preventive diagnosis [10].

In recent years, attention in veterinary cardiology has shifted toward the search for serum or plasma biomarkers capable of reflecting myocardial damage at the molecular level long before the mechanical function of the heart is altered [11]. Among these, cardiac troponin I (cTnI) is widely used as a sensitive and relatively specific biomarker of cardiomyocyte injury in dogs and cats [12]. Conversely, N-terminal pro-B-type natriuretic peptide (NT-proBNP) is widely used as a marker of ventricular wall biomechanical stress, with circulating concentrations increasing in response to cardiomyocyte stretch caused by pressure or volume overload [13].

Finally, and more innovatively, cardiac fatty acid-binding protein (h-FABP) has begun to be investigated within the veterinary field [14]. It is a small cytosolic protein involved in long-chain fatty acid metabolism in the myocardium. Because of its low molecular weight and cytosolic localization, h-FABP may be rapidly released into the circulation following acute ischemic or toxic myocardial injury, potentially before troponin release [15].

Although h-FABP has been proposed as a rapidly released biomarker of myocardial injury, its usefulness for monitoring doxorubicin-associated myocardial damage in dogs remains uncertain, particularly when samples are obtained during clinically practical monitoring windows rather than immediately after drug administration. In addition, the relationship between h-FABP, established cardiac biomarkers, echocardiographic variables, and Holter-derived indices has not been sufficiently characterized in dogs receiving doxorubicin. Therefore, the objective of this exploratory study was to describe changes in h-FABP, cTnI, NT-proBNP, conventional echocardiographic variables, and Holter-derived heart rate variability parameters in dogs with hemangiosarcoma before doxorubicin treatment and one week after the third dose.

## 2. Materials and Methods

### 2.1. Animal Population

This prospective, longitudinal, observational pilot study with repeated measures was conducted at the Veterinary Teaching Hospital of the University of Extremadura, Spain. The study used a paired before–after design, in which each dog served as its own control.

Client-owned dogs with hemangiosarcoma presented to the Oncology Service of the Veterinary Teaching Hospital of the University of Extremadura were enrolled between October 2023 and June 2025. Data collection was conducted from October 2023 to June 2025. The study was exploratory and descriptive in nature and was designed to evaluate temporal changes in cardiac biomarkers, conventional echocardiographic variables, and Holter-derived parameters during doxorubicin treatment.

The experimental protocol involving canine patients and the processing of their biological samples was approved by the Animal Experimentation Ethics Committee of the University of Extremadura under registration number 158/2025 on 12 June 2025, in accordance with Royal Decree 53/2013 (Spain). All procedures were conducted in strict accordance with institutional animal welfare guidelines, and written informed consent was obtained from all owners prior to patient recruitment.

Baseline demographic and oncological characteristics of the enrolled dogs are summarized in Table 1. All dogs received doxorubicin administered intravenously at a dose of 30 mg/m^2^ every 3 weeks. No dose reductions, treatment delays, or deviations from the planned 3-week dosing interval occurred before the T1 evaluation and no additional chemotherapeutic with recognized cardiotoxic potential was administered during the study period.

Study time points were standardized for all dogs. T0 was defined as immediately before the first doxorubicin administration, whereas T1 was defined as 1 week after the third doxorubicin dose. Thus, all dogs had received a cumulative doxorubicin dose of 90 mg/m^2^ at T1. Blood sampling, complete echocardiographic examination, and 24-h Holter monitoring were performed at both T0 and T1.

Inclusion criteria required all animals to have normal baseline cardiac function, as determined by physical examination, screening electrocardiography, and complete echocardiographic evaluation before treatment initiation. Exclusion criteria included chronic degenerative valvular disease, primary cardiomyopathy, concomitant renal insufficiency, or previous exposure to potentially cardiotoxic therapies.

No predefined operational endpoint of cardiotoxicity was established in this exploratory study. Therefore, cardiotoxicity was not classified as present or absent based on a diagnostic reference standard such as a predefined reduction in fractional shortening or ejection fraction, biomarker threshold, arrhythmia burden, clinical signs, or long-term follow-up evidence of cardiac dysfunction. Consequently, sensitivity, specificity, predictive values, diagnostic utility, and early diagnostic performance could not be assessed. The analyses were limited to describing within-dog changes in biomarkers, echocardiographic variables, arrhythmic events, and Holter-derived HRV parameters between T0 and T1.

### 2.2. Cardiac Biomarkers

Blood samples were obtained via jugular or cephalic venipuncture at two critical time points in the study: immediately prior to initiating the first doxorubicin infusion (Time 0 or T0) and following three doxorubicin doses, at a cumulative dose of 90 mg/m^2^ (Time 1 or T1). Blood was collected in clot-activator tubes for serum separation or EDTA tubes for plasma separation, in accordance with the requirements of the analytical kits. The samples were immediately centrifuged at 1000 rpm for 10 min; the supernatant was aliquoted and stored at −80 °C until collective processing.

Quantification of cardiac troponin I (cTnI) was performed using a high-sensitivity quantitative immunoassay (Idexx^®^ Laboratories, Madrid, Spain). According to the laboratory reference interval, cTnI concentrations between 0 and 0.06 ng/mL were considered within the reference limits. NT-proBNP was determined using a commercial ELISA assay validated for the canine species (Idexx^®^ Laboratories, Madrid, Spain). For clinical interpretation, NT-proBNP concentrations < 900 pmol/L were considered associated with a low probability of congestive heart failure, values between 900 and 1800 pmol/L were considered equivocal, and values > 1800 pmol/L were considered associated with a high probability of congestive heart failure. Meanwhile, h-FABP levels were measured using a commercial canine h-FABP ELISA kit validated for canine samples (Ref. MBS2603170-96, MyBioSource, Inc., San Diego, CA, USA). The manufacturer-reported standard curve range is 0.1–40 ng/mL, with an analytical sensitivity of 0.048 ng/mL, an intra-assay coefficient of variation <8%, and an inter-assay coefficient of variation < 10%. Absorbance was measured at 450 nm using a Biochrom Asys UVM 340 microplate reader (Biochrom Ltd., Cambridge, UK). Although this assay is validated for canine samples, a clinical reference interval for h-FABP has not yet been established in dogs. These reference intervals and clinical decision thresholds were used for descriptive interpretation only and were not used to define cardiotoxicity in the present exploratory study.

Cardiac troponin I and NT-proBNP analyses were performed by IDEXX Laboratories (Madrid, Spain). Detailed analytical performance data, including assay-specific limits of detection and intra- and inter-assay coefficients of variation, were not available to the investigators from the external laboratory and were therefore not independently reported.

Samples were aliquoted and stored at −80 °C for five months before analysis. Each sample underwent one freeze–thaw cycle. Samples were analyzed in duplicate, and the analyst was not blinded to the sampling time point.

All comparisons between T0 and T1 were performed using paired analyses, with each dog serving as its own control. Thus, the statistical tests evaluated within-dog changes in biomarker concentrations and cardiac variables rather than comparisons between independent group-level averages.

### 2.3. Echocardiographic Protocol

All echocardiographic studies were performed without sedation to avoid hemodynamic interference with cardiac variables. The animals were placed in right and left lateral recumbency on a specialized echocardiography table, utilizing an ultrasound machine equipped with multifrequency sector probes adapted to the weight of each patient. Images were captured and analyzed by a single experienced operator using a commercial ultrasound system (Vivid E95 echocardiograph, GE HealthCare, Chicago, IL, USA) and EchoPAC softwarev204 (GE HealthCare, Chicago, IL, USA). Echocardiographic examinations were performed at the two predefined study time points: T0, immediately before the first doxorubicin administration, and T1, 1 week after the third doxorubicin dose.

The evaluated variables included fractional shortening (FS, %), calculated in M-mode from the left ventricular short-axis view at the level of the papillary muscles; ejection fraction (EF, %), estimated using Simpson’s method; left atrium-to-aorta ratio (LA/Ao), obtained from the two-dimensional short-axis view at the cardiac base at the onset of diastole; and E-point septal separation (EPSS, cm), measured in M-mode in the short- or long-axis view as the minimum distance between the E-point of the anterior mitral valve leaflet and the interventricular septum. Left ventricular end-diastolic and end-systolic diameter indices (LVDd index and LVDs index) were calculated by normalizing the corresponding ventricular diameters to body weight using standard allometric formulas. End-systolic volume index (ESVI, mL/m^2^) was derived from the end-systolic volume obtained using Simpson’s method and indexed to body surface area.

### 2.4. Holter Monitoring Protocol

Immediately following the clinical and echocardiographic evaluations at T0 and T1, 24-h continuous electrocardiographic monitoring was performed using a Holter system (SEER 1000 model, GE HealthCare, Chicago, IL, USA), with data analyzed using CardioDay^®^ V2.5 software (GE HealthCare, Chicago, IL, USA). Selective shaving of the lateral thoracic regions was performed, and adhesive electrodes were secured. The recording equipment was stabilized on each patient using a commercial Holter vest (HeartVets, Specialist Veterinary Cardiology Consultancy Ltd., Exeter, UK).

Analyzed recordings were required to have a minimum of 18 h of viable, high-quality, artifact-free data. Data were processed using specialized software, where all QRS complexes were automatically classified and visually verified to exclude ectopic beats and technical artifacts.

Arrhythmic events such as ventricular premature complexes (VPCs), doublets, triplets, bigeminy, and ventricular tachycardia were assessed.

To assess cardiac autonomic activity, time-domain heart rate variability (HRV) parameters were determined from the normal R-R intervals of sinus origin. These metrics included the standard deviation of all normal RR intervals (SDNN), the standard deviation of the averages of RR intervals in 5-min segments (SDANN), the root mean square of successive differences between adjacent RR intervals (rMSSD), the percentage of successive RR intervals differing by more than 50 ms (pNN50), and the mean of the SDNN indexes for all 5-min intervals (SDNN Ind).

Furthermore, frequency-domain parameters were evaluated, including very low frequency (VLF), ultra-low frequency (ULF), low frequency (LF), high frequency (HF), and the LF/HF ratio, which estimates the balance between the sympathetic and parasympathetic branches of the autonomic nervous system. These indices allow for the early identification of autonomic tone alterations that could precede structural myocardial dysfunction.

### 2.5. Statistical Analysis

Data analysis was performed using SPSS statistical software 31.0 (IBM Corp., Armonk, NY, USA). Given the exploratory nature of the study and the small sample size (*n* = 14), Shapiro–Wilk normality testing was used to assess data distribution but was interpreted cautiously and was not used as the sole basis for statistical inference. Variables with non-normal paired distributions were summarized as median and interquartile range (IQR) and compared between T0 and T1 using the Wilcoxon signed-rank test. For these variables, the Hodges–Lehmann estimate of the paired median difference (T1 − T0) and its 95% confidence interval were reported. Variables considered appropriate for parametric analysis were expressed as mean ± standard deviation (SD) and compared between T0 and T1 using the paired Student’s *t*-test. *p*-value < 0.05 was considered statistically significant.

Given the multiplicity of comparisons, the Benjamini–Hochberg procedure was applied to the cardiac biomarker and echocardiographic outcome families, in which nominally significant findings were observed. For the remaining exploratory analyses, unadjusted *p*-values are reported because no nominally significant differences were identified. Statistical significance for biomarker and echocardiographic outcomes was interpreted according to the adjusted *p*-values. All analyses were considered exploratory.

## 3. Results

### 3.1. Population and Baseline Characteristics

Fourteen dogs with hemangiosarcoma completed the evaluations at both study time points. The cohort included six females and eight males, with a median age of 10 years (range, 6–13 years). Visceral hemangiosarcoma was recorded in 11/14 dogs (78.6%), whereas three dogs (21.4%) had non-visceral primary tumors. Five dogs were classified as clinical stage I, five as stage II, and four as stage III. Surgical excision of the primary tumor was performed in 9/14 dogs (64.3%).

### 3.2. Evaluation of Cardiac Biomarkers

**Figure 1:**
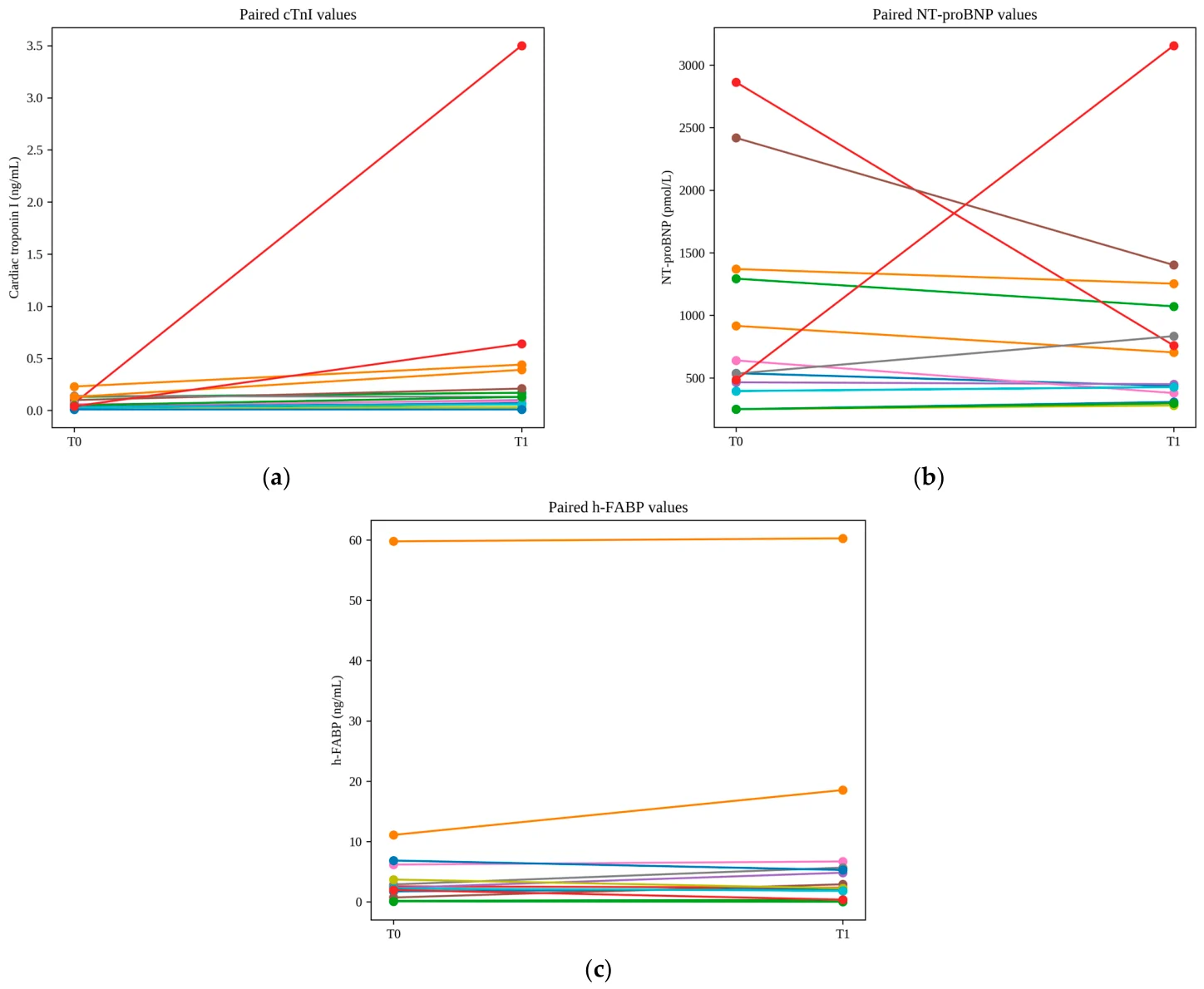
Individual paired biomarker values in dogs before doxorubicin administration (T0) and one week after the third doxorubicin dose (T1). (**a**) Cardiac troponin I (cTnI), (**b**) NT-proBNP, and (**c**) h-FABP. Each point represents one dog, and lines connect paired measurements from the same dog. Paired comparisons were performed using the Wilcoxon signed-rank test. For NT-proBNP, baseline values reported as <250 pmol/L were plotted as 250 pmol/L.

**Figure 2:**
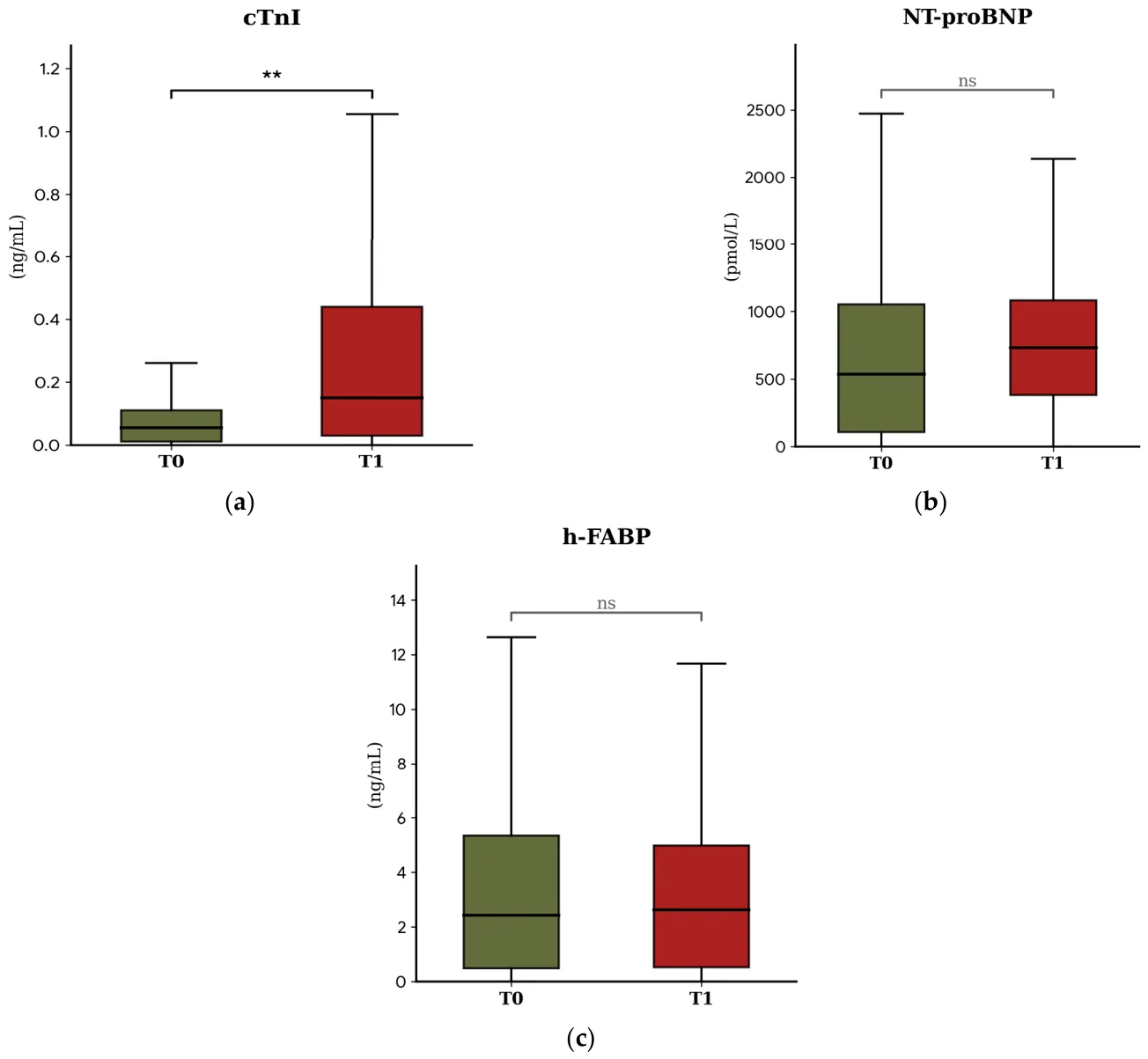
(**a**) cTnI, (**b**) NT-proBNP and (**c**) h-FABP of dogs before (T0) and after three doses of DOX (T1). Data are presented as boxes and whiskers. Each box includes the interquartile range, the line within a box represents the median and the whiskers represent values within 1.5× interquartile range. **: statistically significant difference (*p* < 0.01); ns: not statistically significant.

The Shapiro–Wilk test indicated a non-normal distribution for all three cardiac biomarkers; therefore, paired comparisons between T0 and T1 were performed using the Wilcoxon signed-rank test. Individual paired values for cTnI, NT-proBNP, and h-FABP are presented in Figure 1. The distribution of biomarker concentrations at each time point is presented in Figure 2.

Cardiac Troponin I (cTnI) demonstrated a statistically significant increase from baseline to the post-chemotherapy period. Median cTnI concentration increased from 0.05 ng/mL (IQR, 0.10 ng/mL) at T0 to 0.15 ng/mL (IQR, 0.41 ng/mL) at T1 resulting in a *p* = 0.002. The Hodges–Lehmann estimate of the paired difference (T1 − T0) was 0.130 ng/mL (95% CI, 0.035 to 0.545 ng/mL; adjusted *p* = 0.006). For descriptive context, using the laboratory upper reference limit of 0.06 ng/mL, cTnI concentrations exceeded this value in 6/14 dogs (42.9%) at T0 and in 11/14 dogs (78.6%) at T1. At the individual level, cTnI concentrations increased in 12/14 dogs and remained unchanged in 2/14 dogs; no individual decrease was observed.

In contrast, NT-proBNP concentrations did not differ significantly between time points. The median at T0 was 536 pmol/L (IQR: 946) and at T1 was 731.5 pmol/L (IQR: 702.25), resulting in a *p* = 0.87. The Hodges–Lehmann estimate of the paired difference (T1 − T0) was −29 pmol/L (95% CI, −478.5 to 227 pmol/L). For descriptive interpretation, NT-proBNP concentrations were classified according to the laboratory interpretive thresholds. At T0, 9/14 dogs had concentrations <900 pmol/L, 3/14 had concentrations between 900 and 1800 pmol/L, and 2/14 had concentrations >1800 pmol/L. At T1, the corresponding numbers were 10/14, 3/14, and 1/14 dogs, respectively. These thresholds were used for descriptive purposes only and were not used to define cardiotoxicity.

Values for cardiac fatty acid-binding protein (h-FABP) showed no statistically significant variations between the two periods. The baseline median at T0 was 2.43 ng/mL (IQR: 4.86) and at T1 stood at 2.62 ng/mL (IQR: 4.46), *p* = 0.27. The Hodges–Lehmann estimate of the paired difference (T1 − T0) was 0.410 ng/mL (95% CI, −0.550 to 1.650 ng/mL). No established clinical reference interval or decision threshold was available for the h-FABP assay used in this study. Therefore, the number of dogs exceeding a reference limit could not be reported.

### 3.3. Renal and Hematological Parameters

To provide additional clinical context for the interpretation of cardiac biomarker findings, serum creatinine and hematological variables related to anemia were evaluated at T0 and T1.

Serum creatinine concentrations were available at T0 for all 14 dogs and at both T0 and T1 for 13 dogs. Among dogs with paired measurements, median creatinine concentration was 1.09 mg/dL (IQR, 0.10) at T0 and 1.07 mg/dL (IQR, 0.20) at T1, with no statistically significant difference between time points (*p* = 0.36). Creatinine data were not available at T1 for one dog.

Hematocrit and hemoglobin concentrations were also assessed to evaluate potential changes related to anemia or hemorrhage during the study period. At T0, median hematocrit and hemoglobin concentrations were 40.4% and 14.6 g/dL, respectively. At T1, median hematocrit and hemoglobin concentrations were 37.8% and 12.9 g/dL, respectively. Among dogs with paired hematological measurements, no statistically significant differences were detected in hemoglobin (*p* = 0.11) or hematocrit (*p* = 0.25) between T0 and T1.

### 3.4. Echocardiographic Findings

The evaluated echocardiographic variables showed non-normal distributions; therefore, paired comparisons between T0 and T1 were performed using the Wilcoxon signed-rank test. EPSS increased from a median of 3 mm (IQR, 3 mm) at T0 to 4 mm (IQR, 3.2 mm) at T1. The Hodges–Lehmann estimate of the paired difference (T1 − T0) was 0.5 mm (95% CI, 0.00 to 1.5 mm; raw *p* = 0.04). However, after adjustment for multiple comparisons using the Benjamini–Hochberg procedure, this difference was no longer statistically significant (adjusted *p* = 0.20).

Systolic functional variables such as FS (T0: 33.5%, IQR, 11.0% vs. T1: 37.5%, IQR, 9.0%; Hodges–Lehmann difference, 1.0 percentage point; 95% CI, −3.0 to 6.5; *p* = 0.57) and EF (T0: 64.5%, IQR, 11.5% vs. T1: 58.0%, IQR, 24.5%; Hodges–Lehmann difference, −4.0 percentage points; 95% CI, −10.5 to 2.0; *p* = 0.22) did not show statistically significant differences between time points.

Likewise, the LA/Ao ratio remained similar between T0 and T1 (T0: 1.14, IQR, 0.31 vs. T1: 1.10, IQR, 0.30; Hodges–Lehmann difference, −0.05; 95% CI, −0.125 to 0.085; *p* = 0.36), as did the left ventricular diastolic diameter index (LVDd index; T0: 1.35, IQR, 0.28 vs. T1: 1.35, IQR, 0.23; Hodges–Lehmann difference, −0.013; 95% CI, −0.081 to 0.050; *p* = 0.68).

Among the 13 dogs with paired measurements, the LVDs index also showed no statistically significant difference between T0 and T1 (T0: 0.855, IQR, 0.23 vs. T1: 0.826, IQR, 0.18; Hodges–Lehmann difference, −0.018; 95% CI, −0.094 to 0.054; *p* = 0.55). Similarly, ESVI did not differ significantly between time points (T0: 11.0, IQR, 24.0 vs. T1: 18.0, IQR, 19.5; Hodges–Lehmann difference, 1.0; 95% CI, −2.0 to 4.5; *p* = 0.40). Overall, no echocardiographic variable showed a statistically significant difference between T0 and T1 after adjustment for multiple comparisons.

### 3.5. Holter Monitoring

Twenty-four-hour Holter recordings obtained at T0, immediately before the first doxorubicin administration, and at T1, 1 week after the third doxorubicin dose, were analyzed (Table 2 and Table 3).

Complete paired 24-h Holter recordings were available for 11 dogs at both T0 and T1. Ventricular arrhythmic events were infrequent, with most dogs showing no events during the recording period. No dog recorded more than three events within any individual ventricular arrhythmia category at either time point. Therefore, arrhythmic outcomes are presented descriptively as the number of dogs with 0, 1, 2, or 3 events per 24-h recording, together with the total number of events recorded for each arrhythmia category (Supplementary Table S1). No formal inferential comparisons were performed.

#### 3.5.1. Heart Rate Variability Parameters

Although spectral and temporal HRV analyses showed contrasting biological distributions, they yielded consistent clinical conclusions.

##### Time-Domain Parameters

Due to the non-normal distribution of the data, the Wilcoxon signed-rank test was applied, showing that no significant differences existed in the chronological variability parameters evaluated between T0 and T1 (all *p* > 0.05).

##### Frequency-Domain Parameters

In contrast to the previous variables, the spectral frequency components of HRV followed a normal distribution in the Shapiro-Wilk test. Therefore, the parametric paired Student’s *t*-test was applied. No evidence of a difference in frequency-domain HRV parameters between T0 and T1 was detected at the evaluated time point (all *p* > 0.05).

## 4. Discussion

Monitoring doxorubicin-induced cardiotoxicity remains one of the most complex clinical challenges within comparative veterinary oncology and cardiology, and the present study provided mixed findings that require cautious interpretation.

In this exploratory cohort, cTnI concentrations were higher at T1 than at T0. This statistically significant within-dog increase represents a detectable biomarker change at the evaluated time point. However, because the study did not include a control group, a predefined cardiotoxicity endpoint, or a diagnostic reference standard, it cannot establish whether this increase reflects doxorubicin-induced myocardial injury, nor can it determine the sensitivity, specificity, diagnostic accuracy, or prognostic value of cTnI. Similarly, the absence of statistically significant changes in conventional echocardiographic variables does not establish that biomarker changes preceded functional cardiac dysfunction.

This behavioral pattern of cTnI is strongly supported by the scientific literature in veterinary medicine. Serial monitoring of cTnI in canine patients diagnosed with lymphoma or osteosarcoma receiving doxorubicin chemotherapy demonstrated a significant increase in the concentrations of this biomarker following drug administration. A critical principle highlighted by these authors is that, in the group of dogs that subsequently developed clinical anthracycline cardiomyopathy, serum cTnI concentrations were already markedly elevated before clinical signs were observed. This consolidates cTnI as a sentinel marker of cardiomyocyte toxicity, which occurs directly due to severe oxidative stress inflicted by doxorubicin upon cardiac tissue [16]. In our study, statistically significant differences were observed following the administration of the third doxorubicin dose, reaching a cumulative dose of 90 mg/m^2^. These findings closely align with previous reports in the literature, in which canine patients underwent serial evaluations under a protocol of 30 mg/m^2^ of doxorubicin administered every three weeks. In that study, assessments and sampling performed at week 9, with cumulative dose of 90 mg/m^2^, revealed statistically significant increases in serum cardiac troponin I (cTnI) concentrations compared to day 0 baseline values and a healthy control group [17]. Our results are consistent with those of another prospective pilot study where dogs treated with DOX in which the cTnI levels were significantly affected by treatment, exceeding the reference ranges in 11 out of 13 dogs at least at once during the therapeutic protocol. The clinical relevance of the observed increase should nevertheless be interpreted cautiously. Although a greater number of dogs exceeded the laboratory upper reference limit at T1 than at T0, the median cTnI concentration increased from 0.05 ng/mL to 0.15 ng/mL, and the observed values were markedly lower than those reported in dogs with lethal doxorubicin-induced cardiotoxicity. In the study by Selting et al., cTnI concentrations ranged from 9.30 to 26.38 ng/mL in dogs that developed acute cardiac failure [16]. Therefore, the increase observed in the present cohort should be interpreted as a statistically detectable within-dog biomarker change at the evaluated time point, rather than evidence of severe or clinically overt doxorubicin-associated cardiotoxicity. Because no predefined cardiotoxicity endpoint or long-term cardiac outcome was available, the prognostic significance of this finding cannot be determined.

Several clinical studies in humans highlight the potential of NT-proBNP as a biomarker for doxorubicin-induced cardiotoxicity [18,19,20]. Specifically, elevations in this marker have been reported before clear echocardiographic signs of left ventricular systolic dysfunction in some studies [18,20]. In contrast, other authors observed that NT-proBNP did not reliably identify cardiac alterations following anthracycline treatment, a discrepancy that may be related to differences in study populations, diagnostic protocols, and assessment techniques [21]. In the present study, NT-proBNP concentrations did not show a statistically significant difference between T0 and T1. This finding should be interpreted as no evidence of a detectable difference at the evaluated time point, rather than evidence that NT-proBNP is not useful for monitoring anthracycline-associated cardiac effects.

The absence of a detectable change after a cumulative doxorubicin dose of 90 mg/m^2^ may be related to the early treatment stage, the timing of sampling, or the absence of sufficient ventricular wall stress to produce measurable NT-proBNP changes. Unlike cTnI, which is generally interpreted as a marker of cardiomyocyte injury, NT-proBNP is associated with myocardial wall stress and may become more informative when structural or functional alterations are present [10]. However, the present study cannot determine the diagnostic sensitivity, comparative usefulness, or clinical superiority of NT-proBNP and cTnI for the detection of subclinical doxorubicin-associated cardiac effects.

The behavior of h-FABP in the present study deserves careful interpretation. h-FABP did not show a statistically significant difference between T0 and T1, and this finding should be interpreted within the specific sampling window evaluated rather than as evidence against its potential usefulness as a biomarker. Differences in the release kinetics of h-FABP and cTnI may be relevant when interpreting these results.

h-FABP is a small (15 kDa), soluble cytosolic protein freely distributed within the cardiomyocyte cytoplasm [22]. Following an acute cellular insult, such as myocardial infarction or severe ischemia, h-FABP may diffuse rapidly across damaged cell membranes and has been reported to reach peak plasma concentrations within 4 to 6 h after injury [23]. In contrast, troponins are integral components of the cardiomyocyte contractile apparatus. Although a small fraction of cTnI is present in the cytosol, most is released more gradually as structural sarcomeric disruption occurs, potentially resulting in a more prolonged increase in circulating concentrations [11].

Doxorubicin-associated myocardial injury is generally considered a progressive and cumulative toxic process rather than a single acute ischemic event [23]. Therefore, because h-FABP was measured one week after the third doxorubicin administration, the present study cannot distinguish between two possible explanations for the absence of a detectable change at T1. First, h-FABP may not have increased in response to doxorubicin-associated myocardial injury at the cumulative dose and time point evaluated. Alternatively, a transient post-infusion increase may have occurred but may not have been detected because sampling was performed outside the period of greatest circulating concentration. Thus, the absence of a significant h-FABP change at T1 should be interpreted as a time-point-specific finding rather than as evidence that h-FABP does not respond to doxorubicin-associated myocardial injury.

Serum creatinine concentrations were assessed at T0 and T1 and did not show a significant change between time points. This finding does not suggest an evident change in renal function based on creatinine during the study period; however, creatinine alone does not provide a complete assessment of renal function. Therefore, although renal clearance may contribute to the temporal profile of h-FABP, the present data cannot establish the role of renal clearance in explaining the observed h-FABP results. Additional studies including serial post-infusion sampling and more comprehensive renal assessment are needed to clarify the temporal behavior and clinical relevance of h-FABP in dogs receiving doxorubicin.

Nevertheless, the release kinetics of H-FABP may also differ in more advanced stages of DOX-induced cardiotoxicity. Although the cumulative doses evaluated in the present study may not have been sufficient to induce persistent biomarker elevation, more severe myocardial injury and cardiac remodeling could potentially result in sustained h-FABP release. As anthracycline-induced cardiotoxicity progresses toward chronic myocardial dysfunction and, ultimately, congestive heart failure, ongoing cardiomyocyte damage may prolong the presence of H-FABP in the circulation, thereby extending its diagnostic utility beyond the immediate post-injury period. This hypothesis is supported by previous studies demonstrating significantly higher plasma h-FABP concentrations in dogs with dilated cardiomyopathy (DCM) and myxomatous mitral valve disease (MVD) than in healthy controls, with the greatest elevations observed in animals with more advanced disease (ACVIM stage C versus stage B) [24]. In addition, the absence of a marked or sustained increase in h-FABP concentrations may be related to the relatively low cumulative doxorubicin dose and the single sampling time point evaluated in this study.

When analyzing conventional echocardiographic variables in our cohort following the administration of the third cycle of doxorubicin (T1), relative stability of the evaluated mechanical and volumetric indices was observed. Neither Simpson-derived ejection fraction (EF) nor fractional shortening (FS) showed statistically significant reductions compared with their respective baseline values (T0). Likewise, no statistically significant changes were detected in the LA/Ao ratio, left ventricular diastolic diameter index (LVDd index), left ventricular systolic diameter index (LVDs index), or end-systolic volume index (ESVI). These data contrast with the findings from a recent review of 15 investigations comprising a total of 189 dogs receiving doxorubicin protocols, which identified reductions in systolic function as the principal echocardiographic change among studies reporting echocardiographic alterations. Specifically, doxorubicin was associated with a global mean reduction of 21.24% in EF, and clinical congestive heart failure was reported in 6 of the 9 studies evaluating intravenous doxorubicin administration [25]. 

The apparent contrast between the functional decline described in previous studies and the absence of detectable changes in EF, FS, LVDd index, LVDs index, and ESVI in our cohort may be related to differences in treatment duration, cumulative dose, and timing of assessment. Anthracycline-associated cardiac effects are generally considered dose-dependent and cumulative, and more marked structural or functional abnormalities may occur after higher cumulative doses or more prolonged treatment protocols. In many of the studies included in the aforementioned meta-analysis, longer-duration protocols or higher cumulative doses were evaluated, including experimental regimens with up to 12 administrations [25]. In contrast, the temporal window assessed in the present study, one week after the third dose of doxorubicin, represents an early treatment stage. Therefore, the absence of statistically significant changes in conventional echocardiographic indices at this time point should not be interpreted as evidence that subtle or delayed myocardial dysfunction was absent. Although cTnI showed a statistically detectable within-dog increase, the study design does not allow conclusions regarding myocardial injury or the temporal relationship between biomarker changes and subsequent echocardiographic dysfunction.

Although a nominal increase in E-point septal separation (EPSS) was observed at T1 relative to T0, this difference was no longer statistically significant after Benjamini–Hochberg adjustment for multiple comparisons. In addition, the absolute difference was small, and no intra- or inter-observer reproducibility analysis was performed. Therefore, the observed change may lie within echocardiographic measurement variability. In the absence of concurrent changes in LVDd index, LVDs index, ESVI, EF, or FS, this finding should not be interpreted as evidence of early ventricular remodeling or clinically relevant systolic dysfunction.

In the present study, electrocardiographic monitoring with 24-h Holter did not evidence statistically significant differences between values obtained prior to treatment (T0) and following the administration of the third cycle of doxorubicin (T1). In detail, ventricular premature complexes (VPCs) showed only a numerical increase in their mean value. Similarly, other ectopic phenomena such as couplets, triplets, episodes of bigeminy, and ventricular tachycardia exhibited marginal, isolated increases, but without statistical differences between both evaluation time points. No evidence of a difference in ventricular arrhythmic events between T0 and T1 was detected at the evaluated time point. However, given the small sample size, between-dog variability, and the single post-treatment assessment, these findings cannot exclude subtle, transient, or later-occurring electrophysiological changes. On the other hand, HRV analysis, considered an indirect reflection of the modulation that the autonomic nervous system (ANS) exerts over the sinoatrial node, also failed to yield statistically relevant variations. When contrasting time-domain parameters, critical variables measuring short-term parasympathetic activity and global variability remained stable in their medians between T0 and T1. An identical pattern of stability was detected in frequency-domain components, including very-low-frequency (VLF), low-frequency (LF), high-frequency (HF) bands, and the LF/HF autonomic balance ratio. Various longitudinal studies indicate that progression toward a pattern of dysautonomia, characterized by a marked drop in HRV and sustained sympathetic hyperactivity, is an eminently cumulative and late phenomenon. Previous investigations demonstrated that statistically significant decreases in HRV indices tend to manifest consistently only when critical thresholds of cumulative doxorubicin doses are reached or exceeded, typically situated above 180–200 mg/m^2^ [10,26]. In our experimental design, oncological patients were evaluated after the third cycle of treatment, equivalent to a moderate cumulative dose of approximately 90 mg/m^2^. Accordingly, no evidence of a difference in time- or frequency-domain HRV parameters was detected at the evaluated time point. These findings should not be interpreted as confirmation of preserved autonomic balance, as subtle or delayed changes cannot be excluded in this small exploratory cohort. Overall, 24-h Holter monitoring did not identify statistically significant differences in arrhythmic events or HRV parameters at the evaluated time point. Nevertheless, it provided complementary information on cardiac rhythm and autonomic variables during the early phase of doxorubicin treatment.

This study has several important limitations. First, the small sample size and the variability observed in several outcomes limit the precision of the estimated changes and the ability to detect small or moderate differences. Second, the absence of a control group prevents separation of treatment-associated changes from temporal variation, tumor-related systemic effects, perioperative changes, or other clinical factors. In dogs with hemangiosarcoma, anemia, hemorrhage, inflammation, metastatic disease, recent surgery, and hemodynamic instability may influence cardiac biomarkers and cardiovascular variables. Although hematocrit, hemoglobin, and serum creatinine were recorded, these factors were not comprehensively characterized or controlled for in the present study.

In addition, no predefined cardiotoxicity endpoint or diagnostic reference standard was used. Consequently, the study cannot determine diagnostic performance, biomarker sensitivity, specificity, prognostic value, or biomarker superiority. h-FABP was measured only one week after the third doxorubicin administration; therefore, a transient early post-infusion increase could not be assessed. Renal assessment was limited to serum creatinine, and more comprehensive variables, including SDMA, urinalysis, and glomerular filtration rate, were not available.

Although recordings with a minimum of 18 h of analysable data were accepted, variation in recording duration may have affected the stability of frequency-domain HRV estimates, particularly ULF and VLF components. Therefore, these findings should be interpreted cautiously.

Although LVDs index and ESVI were included in the revised analysis, strain echocardiography and other comprehensive Simpson-derived ventricular volume indices, including EDVI, were not systematically available. Intra- and inter-observer reproducibility analyses were not performed; therefore, small changes in echocardiographic variables, including EPSS, may lie within measurement variability.

## 5. Conclusions

In this exploratory cohort of dogs with hemangiosarcoma treated with doxorubicin, cTnI concentrations increased after three treatment cycles, indicating a statistically detectable within-dog biomarker change at the evaluated time point. In contrast, h-FABP, NT-proBNP, and conventional echocardiographic variables did not show statistically significant differences between T0 and T1. Ventricular arrhythmic events were infrequent and were described descriptively, whereas Holter-derived HRV variables did not show statistically significant differences between time points. Because h-FABP was measured one week after doxorubicin administration, a transient early post-infusion increase cannot be excluded. The study design does not permit conclusions regarding myocardial injury, diagnostic performance, prognostic value, or the comparative clinical utility of h-FABP, cTnI, or other monitoring variables. Larger controlled studies with serial post-treatment sampling, predefined cardiotoxicity endpoints, comprehensive renal assessment, and appropriate multiplicity adjustment are needed.

## Figures and Tables

**Table 1:** Baseline demographic, clinical, and oncological characteristics of dogs with hemangiosarcoma included in the study.

Dog	Age (Years)	Sex	Breed	Tumor Location	Stage	Surgery
1	8	F	Pug	Liver	I	Yes
2	9	F	Labrador Retriever	Spleen	II	Yes
3	12	M	Mixed breed	Spleen	I	Yes
4	10	M	Mixed breed	Spleen	I	No
5	12	F	Pit Bull	Spleen	III	Yes
6	11	F	Mixed breed	Spleen and liver	III	No
7	13	M	Labrador Retriever	Spleen	II	Yes
8	10	M	Spanish Greyhound	Spleen	II	No
9	10	F	Mixed breed	Spleen	I	No
10	7	M	Mixed breed	Subcutaneous	II *	Yes
11	6	M	Schnauzer	Liver	I	No
12	7	M	Pit Bull	Spleen	II	Yes
13	12	F	Bulldog	Muscular	III *	Yes
14	8	M	Mastiff	Bone	III	Yes

Abbreviations: F, female; M, male. Clinical stage was assigned according to tumor location, presence of hemoperitoneum or tumor rupture, and evidence of distant metastasis. For visceral hemangiosarcoma, stage I indicated localized disease without hemoperitoneum or distant metastasis; stage II indicated hemoperitoneum or tumor rupture without distant metastasis; and stage III indicated distant metastatic disease. * Non-visceral hemangiosarcoma. For non-visceral hemangiosarcoma, stage II corresponded to subcutaneous involvement and stage III to muscular involvement. Surgery indicates whether surgical resection of the primary tumor was performed.

**Table 2:** Time-Domain heart rate variability parameters in dogs before and after three doses of DOX.

Parameter	T0	T1	H–L Difference (T1 − T0)	IC 95%	*p*-Value
SDNN (ms)	233.50 (113.50)	230 (146.25)	+22	−105.5 to 53.5	0.20
SDANN (ms)	129 (63.50)	137 (56.50)	+4.5	−58 to 37.5	0.54
rMSSD (ms)	253 (236)	279.50 (348)	+2.5	−142.5 to 51	0.84
pNN50 (%)	50 (34)	46 (33.75)	+3	−26 to 13	0.61
SDNN Index (ms)	164.50 (91.86)	186 (157.75)	17.5	−74 to 49	0.51

Abbreviations: SDNN, standard deviation of all normal-to-normal (NN) intervals; SDANN, standard deviation of the averages of NN intervals in all 5-min segments; rMSSD, root mean square of successive differences between adjacent normal-to-normal intervals; pNN50, percentage of successive NN intervals differing by more than 50 ms; SDNN index, mean of the standard deviations of all NN intervals in 5-min segments; H–L, Hodges–Lehmann estimate of the paired median difference; CI, confidence interval. Values at T0 and T1 are presented as median (IQR). H–L differences are expressed as T1 − T0. Paired comparisons were performed using the Wilcoxon signed-rank test. A two-sided *p*-value < 0.050 was considered statistically significant.

**Table 3:** Frequency-Domain heart rate variability parameters in dogs before and after three doses of DOX.

Parameter	T0	T1	*p*-Value
VLF (ms^2^)	8071.40 ± 10,730.83	5426.21 ± 4290.58	0.38
ULF (ms^2^)	1.7706 × 10^11^ ± 5.597 × 10^11^	1.811 × 10^11^ ± 4.548 × 10^11^	0.98
LF (ms^2^)	3838.60 ± 5112.84	2471.42 ± 2062.14	0.25
HF (ms^2^)	52,841.00 ± 63,054.27	47,775.60 ± 37,836.85	0.75
LF/HF ratio	0.11 ± 0.09	0.10 ± 0.11	0.92

Abbreviations: VLF, very low frequency; ULF, ultra-low frequency; LF, low frequency; HF, high frequency; LF/HF, low-to-high frequency ratio. Values are presented as mean ± SD.

## Data Availability

The original contributions presented in this study are included in the article/Supplementary Materials. Further inquiries can be directed to the corresponding author.

## Supplementary Materials

The following supporting information can be downloaded at: https://www.mdpi.com/article/10.3390/vetsci13080735/s1, Table S1: title: Distribution of ventricular arrhythmic eventsrecorded during complete paired 24-h Holter monitoring at baseline (T0) and 1 week after the third doxorubicin dose (T1) in 11 dogs.

## Abbreviations

The following abbreviations are used in this manuscript:

ACVIM	American College of Veterinary Internal Medicine
CI	Confidence Interval
CHF	Congestive Heart Failure
cTnI	Cardiac Troponin I
DCM	Dilated Cardiomyopathy
DOX	Doxorubicin
EDTA	Ethylenediaminetetraacetic acid
EF	Ejection Fraction
ELISA	Enzyme-Linked Immunosorbent Assay
EPSS	E-Point Septal Separation
ESVI	End-Systolic Volume Index
FS	Fractional Shortening
HF	High Frequency
h-FABP	Heart-type Fatty Acid-Binding Protein
HRV	Heart Rate Variability
IQR	Interquartile Range
LA/Ao	Left Atrium-to-Aorta ratio
LF	Low Frequency
LF/HF	Low-to-High Frequency ratio
LVDd index	Left Ventricular Diastolic Diameter index
LVDs index	Left Ventricular End-Systolic Diameter Index
MVD	Myxomatous Mitral Valve Disease
NT-proBNP	N-terminal pro-B-type Natriuretic Peptide
pNN50	Percentage of successive NN intervals that differ by more than 50 ms
rMSSD	Root mean square of successive differences between adjacent RR intervals
ROS	Reactive Oxygen Species
SD	Standard Deviation
SDANN	Standard deviation of the averages of NN intervals in all 5-min segments
SDNN	Standard deviation of all normal-to-normal intervals
SDNN Ind	SDNN index
SPSS	Statistical Package for the Social Sciences
ULF	Ultra-Low Frequency
VLF	Very Low Frequency
