# Supplementary material for: Exploratory Evaluation of Cardiac Fatty Acid-Binding Protein (H-FABP), Troponin I, NT-proBNP, Echocardiography, and Holter Monitoring for the Assessment of Doxorubicin-Induced Cardiac Damage in Dogs
Source: Vet Sci. 2026 Jul 24;13(8):735. doi: 10.3390/vetsci13080735 (PMC13517254; doi:10.3390/vetsci13080735)
Supplement: Supplementary file 1 [file vetsci-13-00735-s001.zip › vetsci-4400395-supplementary.pdf]

Supplementary Table S1. Distribution of ventricular arrhythmic eventsrecorded during complete paired 24-hour Holter monitoring at baseline (T0) and 1 week after the third doxorubicin dose (T1) in 11 dogs.

Arrhythmia category	Events per dog over 24 h	T0 n/11 dogs	T1 n/11 dogs
VPCs	0	8/11	5/11
	1	1/11	3/11
	2	2/11	1/11
	3	0/11	2/11
	**Total events recorded**	**5**	**11**
Doublets	0	10/11	8/11
	1	0/11	2/11
	2	1/11	1/11
	3	0/11	0/11
	**Total events recorded**	**2**	**4**
Triplets	0	10/11	8/11
	1	0/11	2/11
	2	1/11	1/11
	3	0/11	0/11
	**Total events recorded**	**2**	**4**
Bigeminy episodes	0	10/11	6/11
	1	0/11	4/11
	2	1/11	1/11
	3	0/11	0/11
	**Total events recorded**	**2**	**6**
Ventricular tachycardia episodes	0	7/11	9/11
	1	3/11	0/11
	2	1/11	1/11
	3	0/11	1/11
	**Total events recorded**	**5**	**5**

Abbreviations: VPCs, ventricular premature complexes. Values in the rows labelled 0–3 represent the number of dogs with the specified number of events recorded during each 24-hour Holter recording. Complete paired 24-hour Holter recordings were available for 11 dogs at both T0 and T1. Total events recorded represents the cumulative number of events within each arrhythmia category across all dogs. Given the low frequency of events and the high proportion of zero values, arrhythmic outcomes are presented descriptively and no formal inferential comparisons were performed.
